# The Effects of Natural Clinoptilolite and Nano-Sized Clinoptilolite Supplementation on Lipid Profile, Food Intakes and Body Weight in Rats with Streptozotocin-Induced Diabetes

**DOI:** 10.15171/apb.2018.025

**Published:** 2018-06-19

**Authors:** Behnoush Hossein-Nia, Sirous Khorram, Hassan Rezazadeh, Abdolrasol Safaiyan, Rafigheh Ghiasi, Ali Tarighat-Esfanjani

**Affiliations:** ^1^School/Faculty of Nutrition and Food Sciences, Tabriz University of Medical Sciences, Tabriz, Iran.; ^2^Experimental Physics, Materials Physics, Materials Science, University of Tabriz, Tabriz, Iran.; ^3^Department of Pharmacology and Toxicology, School of pharmacology, Tabriz University of Medical Sciences, Tabriz, Iran.; ^4^Road Traffic Injury Research Center, Department of Biostatistics and Epidemiology, Tabriz University of Medical Sciences, Tabriz, Iran.; ^5^Physiology Department, Tabriz University of Medical Sciences, Tabriz University of Medical Sciences, Tabriz, Iran.; ^6^Nutrition Research Center, Faculty of Nutrition, Tabriz University of Medical sciences, Tabriz, Iran.

**Keywords:** Diabetes Mellitus, Lipoproteins, Zeolites, Rats, Body weight

## Abstract

***Purpose:*** To determine the effect of natural clinoptilolite (CLN) and nano-sized clinoptilolite (NCLN) on lipid profile, food intakes (FI) and weight changes in streptozotocin (STZ) induced diabetic rats.

***Methods:*** In this experimental study, 36 rats were randomly divided into two groups: diabetic group which was injected STZ (60 mg/kg BW), and a non-diabetic group. Three days after diabetes induction, each of these groups was randomly divided into 3 subgroups of 6 animals ((1) control, (2) 1%/food CLN, (3) 1%/food NCLN). The animals were supplemented for 28 days, starting three days after STZ administration. At the end of the study, blood was drawn for biochemical assays. The weights and FIs of the rats were measured at the beginning and end of each week.

***Results:*** Our findings revealed that there was no significant change in lipid profile, 28 days after administration of STZ in diabetic rats. Low density lipoprotein (LDL) was increased slightly in diabetic rats treated with NCLN without any significant changes in other lipid profile parameters in the other groups. Weight was reduced significantly in diabetic rats. Administration of CLN and NCLN prevented further weight loss in diabetic rats. All groups treated with STZ had higher food intake during the study.

***Conclusion:*** Lack of beneficial changes in lipid profile may be attributed to short study duration, insufficient for appearance of lipid abnormalities. Given the partial improvement in weight status and lack of undesirable effects of clinoptilolite supplementation, further research is recommended in subjects with typ1 diabetes mellitus.

## Introduction

Diabetes mellitus (DM) is a metabolic dysfunction which is associated with hyperglycemia and derangements of macronutrients metabolism^1^ which are the important causes of diabetic complications. Hyperglycemia may modify lipoproteins to forms which are more likely to lead to atherogenesis.^2^ Changes in lipid levels and subsequent complications of lipid metabolism and stress have been identified in DM.^3^ The lipid abnormalities related to DM include high concentration of total cholesterol (TC) and triglyceride (TG) levels.^4^ Besides, reduction in serum levels of high density lipoprotein cholesterol (HDL-C) was observed in diabetic patients. Low-density lipoprotein-cholesterol (LDL-C) levels are often altered in diabetic sufferers as well.^2^

Zeolites, AlO4 and SiO4 tetrahedral, are hydrated natural or synthetic microporous crystals, linked through the common oxygen atoms.^5^ During the last decades, natural zeolites have had a variety of applications in adsorption, catalysis, industry, agriculture, and energy.^6^ Furthermore, zeolites have been reported to possess antioxidant,^7-9^ antitumor,^10,11^ antiviral^12^ and immunomodulatory activities.^5,13^ Several studies have indicated that the zeolites have no significant effects on serum biochemical parameters in supplemented animals.^14-16^ However, findings of some studies have shown beneficial effects of dietary supplementation of zeolite on health, growth and reproduction performance in animals.^17-20^ According to the ion-exchange properties of zeolites, they are particularly suitable for removing heavy metal ions^21^ and also can reduce the harmful effects of ingested toxins.^22^ A hypocholesterolemic effect has also been reported for zeolite^23,24^ Clinoptilolite is a natural zeolite. In comparison with traditional clinoptilolite (CLN), nano-sized CLN (NCLN) is offered to have a high specific surface area. The increase in the catalyst surface area of NCLN leads to an increase in quantity of substance available to react and thus improve the rate of the reaction which might positively affect the mentioned process performance.^25^

The great number of side effects attributed to insulin and other antidiabetic drugs are,^23^ has aroused interest among researchers to search for new sources of natural therapeutic antidiabetic compounds; this is essential for overcoming various secondary complications of diabetes.^24^ Hence, we decided to investigate the effects of NCLN on lipid profile, food intakes (FI) and body weight (BW) of diabetic rats induced with Streptozotocin (STZ).

## Materials and Methods

The natural CLN powder used in this experimental study, was a sodium/potassium CLN with particle size of about 5 micrometers (Afrazand Co., Tehran, Iran). The content of CLN supplements is presented in Table 1. The NCLN particle was produced by glow discharge plasma method, a novel Fe-impregnated nanocatalyst for the heterogeneous fenton process). Particle analysis of the NCLN showed that the size of produced nanoparticles was in the range of 30-40 nm diameters.^25^

**Table 1:** Elemental composition of clinoptilolite and nano-sized clinoptilolite

	**Weight (%)**				**mole/ratio**		
	**Na**	**Al**	**Si**	**K**	**Si/Al**	**Na/Al**	**K/Al**
**CLN**	3.58	7.07	60.33	0.72	8.28	1.25	0.15
**NCLN**	8.86	4.81	44.27	10.94	8.88	4.52	3.23

CLN= clinoptilolite; NCLN= nano-sized clinoptilolite; Na=Sodium; Al=Aluminium; Si= Silicon; K=Potassium Adapted with permission from Table 2 in "Khataee, A., Bozorg, S., Khorram, S., Fathinia, M., Hanifehpour, Y., & Joo, S. W. (2013). Conversion of natural clinoptilolite microparticles to nanorods by glow discharge plasma: a novel Fe-impregnated nanocatalyst for the heterogeneous Fenton process. *Industrial & Engineering Chemistry Research*, *52*(51), 18225-18233."

### Chemicals food Preparation 

Animal feeds were purchased from Behparvar Company (Behparvar Co, Tehran, Iran). Experimental feeds were prepared as powder and were mixed with 1% CLN or 1% NCLN powder. Tap water was added to the mixture of materials and then mixed feeds were turned into pellet form feeds and were kept to dry at room temperature.

### Chemicals

Streptozotocin (STZ. Sigma Chemicals, St. Louis, MO, USA), Diethyl ether and other solvents and buffers (Merck, Germany C.) were used in this project.

### Experimental animals

Six-month old, weight >0.25 kg, healthy male Wistar rats were procured from the laboratory animals unit of the Azad university of Marand branch, Tabriz, Iran. The animals were kept under standard environmental condition (temperature 23 -25°C, and relative humidity of 30–50%) and acclimatized for a period of seven days in the animals' center (Faculty of Pharmacy, Tabriz University of Medical Sciences, Tabriz, Iran) for breeding and maintenance. They were supplied with clean drinking water and pellet. Fundamental guidelines for the care and use of laboratory animals were followed in dealing with the animals.

### Experimental design

In this experimental study, 36 rats were randomly allocated into two groups and then one group was randomly selected as a diabetic group and injected STZ (60 mg/kg BW in 0.1 M sodium citrate buffer, pH=4.5). Rats with blood glucose levels >250 mg/dl were considered as being diabetic. Three days after diabetes induction, each group (diabetic and non-diabetic groups) was randomly divided into three subgroups with 6 animals in each: ((1) control, (2) 1%/food CLN, (3) 1%/food NCLN). The BWs and feed intakes of the rats were recorded at baseline and end of each week using an electronic weighing balance (Model Scout Pro, Ohaus Corporation, USA). At the end of the trial (on 28^th^ day), animals were anesthetized by dimethyl ether and blood samples were collected instantly from the orbital sinus of fasted 12h rats. After blood collection, plasma was separated by centrifugation at 3000 rpm for 10 min and analyzed to estimate the amounts of TC, TG, HDL-C and LDL-C. Blood glucose levels were measured by a glucometer, at the beginning and then at days 14 and 28 of study.

### Biochemical analysis

The serum concentrations of TC, TG, and HDL-C were measured using diagnostic kits (Pars Azmoon kit, IRI) on an automatic analyzer (Abbott, model Alcyon 300, USA). LDL-C was calculated through the Friedewald Equation (Noda *et al*., 2000): [LDL-C] = ([TC] - [TG]/5)

### Statistical methods

The influential variables were adjusted between two groups (Diabetic and Non-Diabetic) in the three sub-groups (Control, CLN, NCLN) on the first day before starting the experiment. Two-way ANOVA was used for comparing TG, TC, LDL-C and HDL-C between diabetic factors (Diabetic and Non-Diabetic) and treatment factors (Control, CLN, NCLN). Mauchly's W test was checked for identity covariance matrix of repeated measure data; then, repeated measure test was used by Minitab Software version 17. Four *p*-values have been presented for our results; the first was *p*-value_Group_ for comparing two diabetics and non-diabetics groups, the second was *p*-value_Treatment_ for comparing treatment factor in each groups, the third was *p*-value_Interaction_ for recognizing interaction effect between diabetic factor and treatment factor and P-value_time_ for comparing variations in five times of intervention. Sidak tests were used for multiple comparisons. The level of significance was set at 0.05 and all results were expressed as Mean±SEM (standard error of mean).

## Results

**Figure 1:**
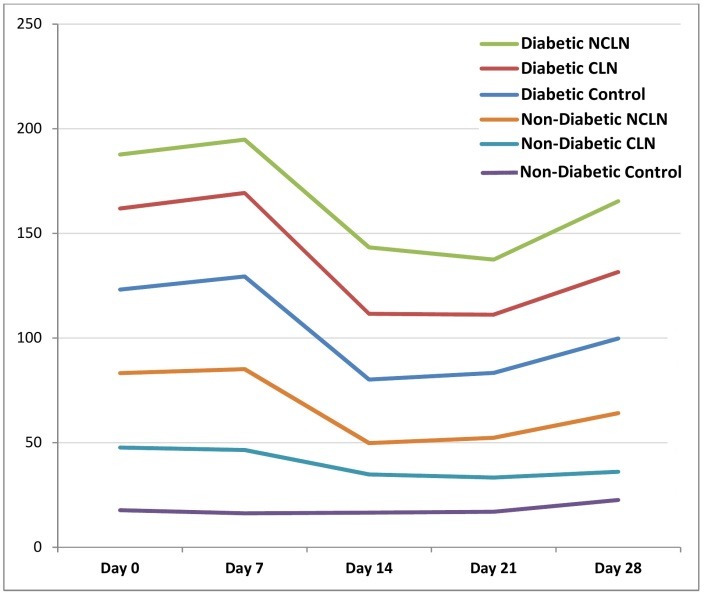
Average daily food intake (g/day) of each group in the beginning and end of each week
(CLN= clinoptilolite; NCLN= nano-sized clinoptilolite)

As shown in Table 2, STZ treatment caused significant weight reduction (Day 14) in rats as compared to normal rats during the study (*p*v<0.001). Nevertheless, the treatment of CLN and NCLN (1% of daily FIs) for 28 days decreased the BW loss as compared to the diabetic untreated control rats. On day 21 of the study, significant BW reduction was observed in CLN treated diabetic group compared to day 0 (*pv*<0.001). Comparison of daily food (g/day) in the study period in each of the studied groups is presented in Figure 1. Across the whole study period, average food intakes were greater in diabetic rats compared to normal rats.

Table 3 presents the mean ±SEM of changes in lipid profile in the experimental groups. No significant changes were observed in HDL, TC, and TG between normal and diabetic groups. Administration of NCLN caused increases in LDL-C, in supplemented diabetic group in comparison with diabetic control group (*pv*=0.042), while supplementation of CLN reduced LDL levels in the diabetic group.

**Table 2:** Effect of CLN and NCLN on body weight in STZ-treated Diabetic Rats

		**Body weight (gr)**					**Repeated Measured Anova P-vlues**			
**Groups (n=6)**	**Treatment**	**Day 0**	**Day 7**	**Day 14**	**Day 21**	**Day 28**	**Diabetes**	**Treatment**	**Interaction**	**Time**
**Non-diabetics**	Control	316.2±10.56	315.2±89.00	316.2±11.17	308.3±14.12	330.2±93.92	<0.001	<0.001	<0.001	<0.001
	CLN	324.3±12.42	322.5±12.75	337.8±11.58	347.6±11.48	349.8±10.86					
	NCLN	332.6±12.29	308.5±11.31	339.8±15.07	335.8±14.17	340.0±14.50				
**Diabetics**	Control	302.6±10.05^*^	306.3±15.97	247.0±80.82	239.0±40.41^*^	238.5±95.26				
	CLN	323.3±11.72	299.8±12.31^*^	280.1±14.70^*^	260.0±14.88*	270.0±94.81				
	NCLN	312.1±13.18	325.0±76.68	268.3±13.15^*^	265.0±13.99^*^	270.8±14.88^*^				

Data are expressed in terms of means ±SEM.; CLN= Clinoptilolite; NCLN= Nano-sized clinoptilolite, *Sidak tests p<0.05 compared to group 2 and time 28

**Table 3:** Effects of CLN and NCLN on lipid profile in STZ-treated Diabetic Rats

**Group**	**Non-diabetic**			**Diabetic**			**Two way Anova P-values**		
**Treatment**	**Control**	**CLN**	**NCLN**	**Control**	**CLN**	**NCLN**	**P**_α_	**P**_β_	**P**_θ_
TG (mg/dL)	46.8±8.66	54.1±3.40	66.8±4.26	51.6±11.51	49.3±11.08	58.0±4.78	0.656	0.232	0.681
TC (mg/dL)	67.2±3.61	65.6±1.28	66.6±4.82	53.3±4.75	62.1±7.21	69.3±2.64	0.189	0.239	0.191
LDL-C (mg/dL)	27.6±4.09	27.5±2.12	22.5±4.51	17±1.86	22.9±2.79	27.9±1.66^*^	0.199	0.552	0.042
HDL-C (mg/dL)	30.2±1.19	27.3±0.84	28.8±0.91	26±3.12	28.5±2.39	29.8±1.62	0.661	0.715	0.28

Data are expressed in terms of means ±SEM; TG: Triglyceride; TC: Total cholesterol; LDL: Low density lipoprotein; HDL: High density lipoprotein; N:Normal rats, CLN: Clinoptilolite; NCLN: Nano-sized clinoptilolite, ANOVA followed by Post-Hoc and Sidak tests. ^*^ p<0.05 compared to group 4; P_α_: diabetes, P_β_: group, P_θ_: interactio

## Discussion

Dietary administration of CLN and NCLN did not significantly effects lipid profile. Supplementation of CLN and NCLN retrieved weight loss in diabetic rats. STZ is a toxin to induce rapid and eternal necrosis of β-cells of the islets of Langerhans, resulting in insulin deficiency and hyperglycemia.^26^ Diabetic hyperlipidemia is a known complication that happens in correlation with severe lack of insulin;^27^ it is associated with hyperglycemia and is characterized by abnormalities in the metabolism of plasma lipids and also changes in lipoproteins.^28^ High incidence of the atherosclerotic disease which is the most common serious and major cause of premature death in diabetic patients has increased interest in study of plasma lipids in diabetes mellitus. Evidence indicates that coronary heart diseases occur as a result of the increases in TG-rich lipoprotein, TG, and cholesterol.^29^

In the present study, STZ injection did induce diabetes in the rats, but failed to cause, clear abnormalities in lipid metabolism of these animals. The normal lipid profile acquired in this study may have been due to the short-term treatment period.^30^ In our study, lipid profile was unaffected by the dietary supplementation of CLN and NCLN, in normal and diabetic rats; however, LDL-C was increased by NCLN administration in diabetic rats. A previous study showed that long-term dietary supplementation of CLN at the rate of 2% resulted in a lower serum Urea-N and cholesterol concentrations in pigs.^31^ Supplementation of CLN for 135 days has also been reported to reduce serum concentration of TC and increase the concentration of TG in normal pigs.^32^ Moreover, Demirel, R *et al*., reported that administration of CLN at three doses (2, 4, and 6%) to male Sprague–Dawley rats resulted in a significant increase in TG and very low density lipoprotein (VLDL) levels, but did not cause any significant changes in cholesterol, HDL-C, and LDL-C levels.^14^ The CLN-supplemented diets tended to produce higher levels of total volatile fatty acids such as propionic acids in beef steers.^33^

There are also reports which indicate adding CLN to diet of broiler increased levels of n-3 fatty acid on the chicken body,^34^ elevated the level of polyunsaturated fatty acids in eggs fat^35^ and had no significant effect on cholesterol, HDL-C, LDL-C, and VLDL in broiler chicken.^36^ The mechanism of these results is not well-comprehended. Considering that zeolite can bind to bile acids in the intestinal tract,^37^ it has been assumed that deconjugation process of bile acids could increase by long-term storage of zeolite in the gastrointestinal tract and also stimulate microbial activity in the small intestine. Moreover, CLN reduces the level of TC in serum trough adsorption of bile salts on its surface in the digestive tract and subsequently increasing the demand for synthesis of bile salts.^32^ Furthermore, natural zeolites have been shown to have the ability to increase the apparent digestibility of the dietary protein in pigs^31^ and total track starch in beef steers.^33^ The hypocholesterolemic effect of CLN‏ can be attributed to alterations in the dietary protein digestibility and in the absorption rate of the dietary cholesterol;^31^ it is notable that dietary protein restriction has revealed hypercholesterolemic effects in growing pigs.^38^

Adding 1% CLN and NCLN to the feed of the diabetic rats, decreased weight loss in diabetic rats, although this improvement was not significant. Weight loss in the diabetic rats is associated with the fact that STZ-induced diabetes leads to catabolism of structural proteins.^39,40^

Studies on several animal species have reported that dietary use of zeolite generally has beneficial effects on BW and FI in animals.^15,18,31^ It has been suggested that administration of dietary zeolite increases hypertrophied functions of intestinal villi and epithelial cells at the duodenum and ileum and leads to further surface area for nutrient absorption and thus improved nutrient digestibility.^18^ Adding zeolite to animal diet has been stated to alter the ionic composition of rumen resulting in an increase in its pH and acetate to propionate ratio and rise rumen fermentation and digestibility of nutrients. Furthermore, absorption attributes of zeolite lead to capture of digestion products of small molecular weight.^41,42^ Administration of zeolite significantly increased digestibility values of crude protein and gross energy, and also increased BW gain and FI in broiler chickens.^17^ Although the exact mechanism of how zeolite affects the growth of animals still can't be clarified, its beneficial effects on growth are attributed to a number of mechanisms, including the binding and/or removing of compounds derived through microbial activity (such as mycotoxins, aflatoxins, ammonia, etc.), positive effects on intestinal microflora, and digestion and secretion of digestive enzymes which leads to better utilization of nutrients.^17,20,22,43-46^

## Conclusion

It can be concluded from the present study that nutritional treatment of NCLN and CLN does not have any effects on lipid profile in diabetic and normal rats; however, it seems to mildly increase LDL-C through interaction of diabetes and NCLN in NCLN- supplemented diabetic rats. Further studies are therefore recommended to give some detailed explanation regarding these results. The results of this study indicate that CLN and NCLN supplementation (1%) does not have beneficial effects on growth of normal rats, but could partially improve the growth in diabetic rats. These findings may be due to factors such as short duration and the low dose of the intervention. It is recommended that these factors are addressed in future studies.

## Acknowledgments

The present study is derived from the thesis of Mrs. Behnoush Hossein-Nia, financially supported by the Vice Chancellor of Research of Tabriz University of Medical Sciences, Tabriz, Iran. At the end, we would like to gratitude all the individuals who helped us in the preparation of this article.

## Ethical Issues

Ethical approval was obtained from the ethics committee of the Tabriz University of Medical Sciences (Tabriz, Iran).

## Conflict of Interest

The authors report no declarations of interest.
